# Polypyrimidine tract-binding protein 3/insulin-like growth factor 2 mRNA-binding proteins 3/high-mobility group A1 axis promotes renal cancer growth and metastasis

**DOI:** 10.1016/j.isci.2024.109158

**Published:** 2024-02-09

**Authors:** Qianqing Wang, Fang Chen, Yu He, Yue Gao, Jiawen Wang, Sufang Chu, Pei Xie, Jiateng Zhong, Haixia Shan, Jin Bai, Pingfu Hou

**Affiliations:** 1 Department of Gynecology Oncology, Xinxiang Central Hospital, The Fourth Clinical College of Xinxiang Medical University, Xinxiang, Henan 453000, China; 2 Cancer Institute, Xuzhou Medical University, Xuzhou, Jiangsu 221004, China; 3 Jiangsu Center for the Collaboration and Innovation of Cancer Biotherapy, Xuzhou Medical University, 209 Tongshan Road, Xuzhou, Jiangsu 221004, China; 4 Department of Oncology, The Affiliated Hospital of Xuzhou Medical University, 99 Huaihai Road, Xuzhou, Jiangsu 221002, China

**Keywords:** Molecular biology, Cell biology, Cancer

## Abstract

Polypyrimidine tract-binding protein 3 (PTBP3) plays an important role in the post-transcriptional regulation of gene expression, including mRNA splicing, translation, and stability. Increasing evidence has shown that PTBP3 promotes cancer progression in several tumor types. However, the molecular mechanisms of PTBP3 in renal cell carcinoma (RCC) remain unknown. Here, tissue microarrays (TMAs) suggested that PTBP3 expression was increased in human RCC and that high PTBP3 expression was correlated with poor five-year overall survival and disease-free survival. We also showed that PTBP3 binds with HMGA1 mRNA in the 3′UTR region and let-7 miRNAs. PTBP3 interacted with IGF2BP3, and the PTBP3/IGF2BP3 axis prevented let-7 mediated HMGA1 mRNA silencing. PTBP3 promotes renal cancer cell growth and metastasis *in vitro* and *in vivo*. Taken together, our findings indicate PTBP3 serves as a regulator of HMGA1 and suggest its potential as a therapeutic agent for RCC.

## Highlights

• PTBP3 promotes renal cancer growth and metastasis via HMGA1
• PTBP3 interacts with IGF2BP3 and binds to HMGA1 3′UTR and let-7 miRNAs
• PTBP3 promotes HMGA1 expressions by stabilizing HMGA1 mRNA

## Graphical abstract

**Figure:**
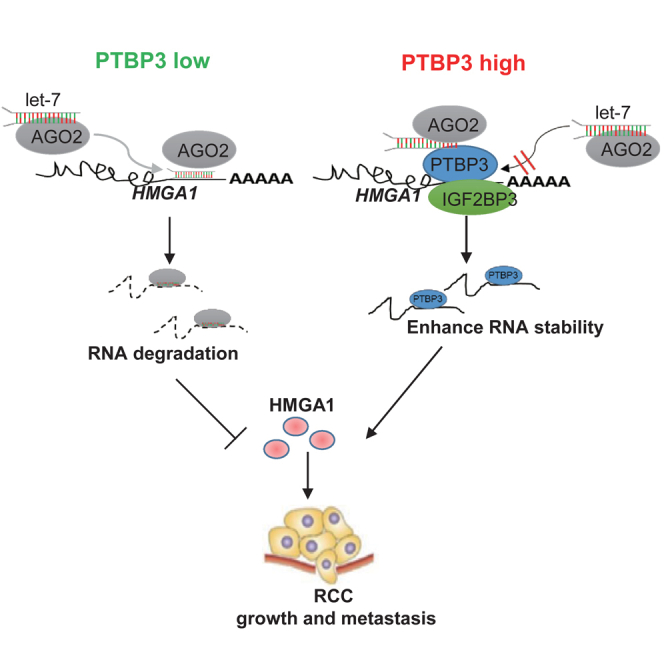

## Introduction

RNA-binding proteins (RBPs) regulate RNA metabolism at the physiological and pathological levels and are involved in the occurrence and development of various diseases.^1^ RBPs engage in almost every step of the post-transcriptional regulation of RNA, including transcription, splicing, modification, intracellular trafficking, translation, and decay.^2^ RBPs are rapidly becoming potential therapeutic targets for various diseases.^3^^,^^4^ Therefore, dissecting RBP-RNA networks may provide new targets for cancer therapy.

Polypyrimdine Tract Binding Protein 3 (PTBP3) belongs to the PTB family. PTBP3 has a structure similar to that of PTBP1, with four RNA recognition domains (RRM1-4).^5^ PTBP3 was originally found to be primarily expressed in hematopoietic cells.^6^ PTBP3 is a cofactor of activation-induced cytidine deaminase (AID) in B cells, PTBP3 can regulate the recognition of AID to the target sites and further affect hyper-IgM syndrome type 2 caused by AID mutation.^7^ PTBP3 is highly expressed in the breast and promotes breast cancer metastasis by increasing the stability of ZEB1 mRNA.^8^ In colorectal cancer, PTBP3 promotes metastasis by enhancing internal ribosome entry segment (IRES)-mediated HIF-1α translation^9^ and promotes cell proliferation by maintaining UBE4A mRNA stability.^10^ The carcinogenic effect of PTBP3 on the cell cycle and growth of gastric cancer cells has also been reported.^11^ PTBP3 promotes hepatocellular cancer by disrupting the splicing balance of NEAT1 and pre-miR-612.^12^ However, the functions and molecular mechanisms by which PTBP3, an RNA-binding protein (RBP), regulates RNA metabolism remain unclear.

High-mobility group A1 (HMGA1) is a nucleoprotein that binds to a ditch in AT-rich DNA strands and regulates the transcriptional activity of genes.^13^ High HMGA1 protein expression has been observed in all neoplastic tissues analyzed, including colon, kidney, breast carcinomas, myeloproliferative neoplasms, and so forth.^14^^,^^15^ HMGA1 regulates malignant features of tumors and is related to resistance to antineoplastic therapy.^16^ HMGA1 can be regulated by several miRNAs, such as let-7, miR-26a, and miR-59, and so forth.^17^^,^^18^^,^^19^ Insulin-like growth factor 2 (IGF2) mRNA-binding proteins (IGF2BPs) can prevent let-7 miRNA target gene silencing, such as HMGA1/2, and so forth.,^20^^,^^21^^,^^22^ suggesting that RBP-miRNA ribonucleoproteins (RNPs) are one of the mechanisms by which RBPs regulate gene expression.

In this study, to determine the mechanism of PTBP3 in RCC, we investigated the expression of PTBP3 in clinical samples of patients with renal cell carcinoma, and analyzed its role and molecular mechanism in regulating the malignant characteristics of RCC. We discovered that PTBP3 was dramatically upregulated in RCC tissues of patients compared to adjacent normal renal tissues, and high PTBP3 expression was correlated with poor five-year overall survival and disease-free survival. Overexpression of PTBP3 promotes renal cancer cell proliferation, migration, and invasion *in vitro* and increases tumor growth and metastasis *in vivo*. Importantly, we found that PTBP3 regulated HMGA1 mRNA and protein expression by increasing the stability of HMGA1. We also showed that IGF2BP3 interacts with PTBP3 and mediates the PTBP3-induced malignant features of RCC. These data provide new insights into the mechanism of RCC tumorigenesis, and support PTBP3 as a potential target for RCC treatment.

## Results

### Polypyrimidine tract-binding protein 3 was upregulated in patients with renal cancer and poor 5-year survival

**Figure 1:**
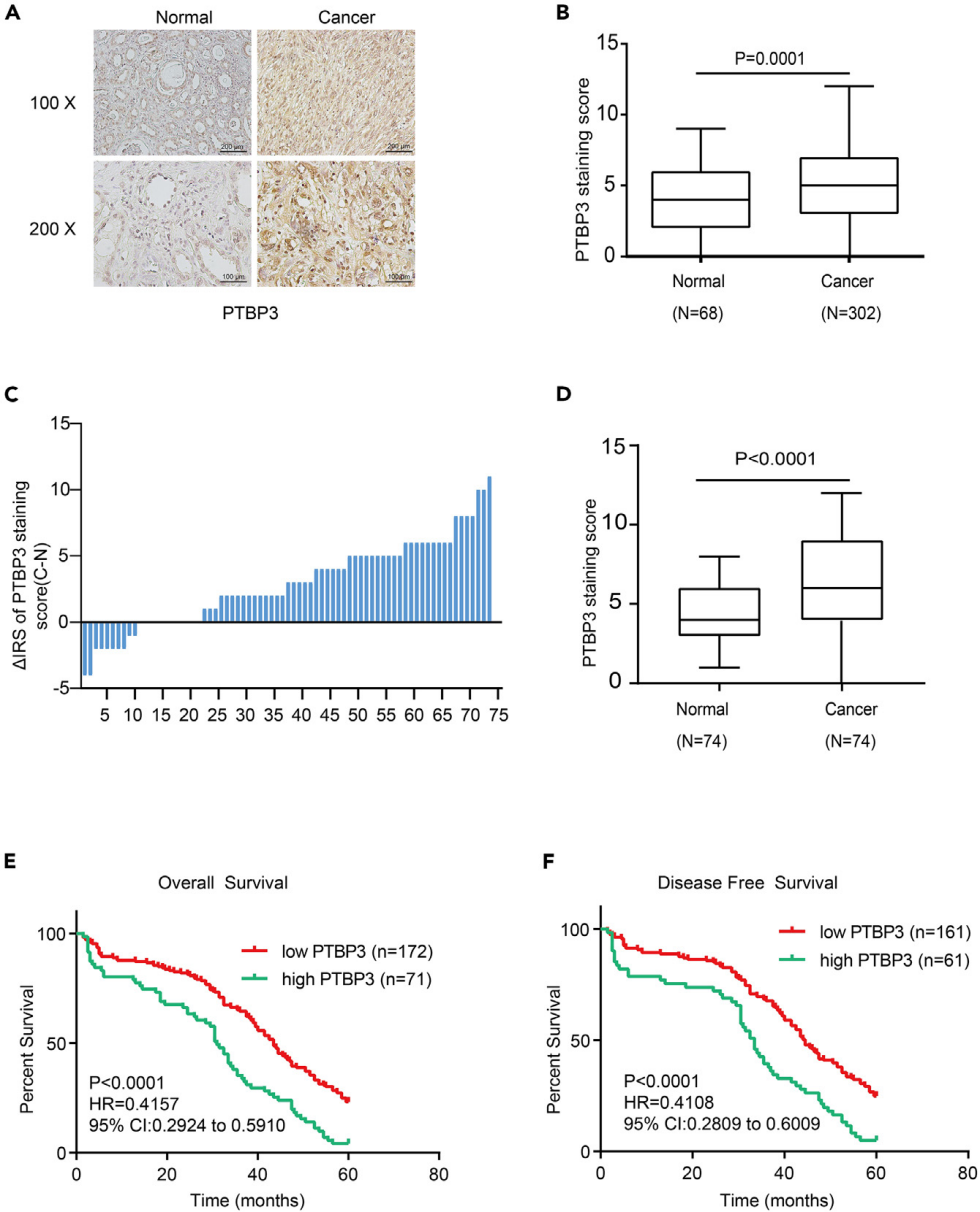
PTBP3 was upregulated in patients with renal cancer and poor 5-year survival (A) Representative immunohistochemical images of PTBP3 protein expression in patients with renal cancer and adjacent non-cancerous tissues. (B) Staining intensities of PTBP3 in 302 cases renal cancer tissues compared with 68 cases normal tissues. Statistical analysis was conducted using a paired t test, p = 0.0001. (C) Staining intensities of PTBP3 in renal carcinoma tissues compared with paired adjacent non-cancerous tissue. N, paired adjacent non-cancerous tissues. C, renal carcinoma tissues (n = 74). (D) Staining intensities of PTBP3 in 74 paired cases of renal cancer tissues and normal tissues. (p < 0.0001). (E and F) Kaplan–Meier survival curves depicting overall survival (n = 243, p < 0.0001) or disease-specific survival of patients with RCC (n = 222, p < 0.0001) stratified by PTBP3 protein expression levels in RCC tissues. Statistical analysis was conducted using log rank test.

To investigate its role in RCC development, we assessed PTBP3 protein expression by IHC in patients with RCC using tissue microarray (TMA) slides, which contained 68 normal renal tissues and 302 renal cancer tissues. The results showed that PTBP3 expression was dramatically upregulated in cancerous tissues compared to normal tissues in patients with RCC (Figures 1A and 1B) (p < 0.001). In the training cohort, TMA slides containing 74 cases of RCC tissues with paired non-tumor tissues, we observed significantly higher PTBP3 expression in tumor tissues than in paired adjacent non-tumor tissues (Figures 1C and 1B). The correlation between PTBP3 expression and clinicopathological characteristics of RCC tissue samples was analyzed (Table 1). Compared with histological grades I and II, the expression of PTBP3 was dramatically increased in stages III and IV (p = 0.036). In addition, high PTBP3 expression positively correlated with distant metastasis (p = 0.028) (Table 1). Kaplan-Meier survival analysis was performed to evaluate the impact of PTBP3 expression on the overall survivals and disease-free survivals in patients with RCC based on the IHC staining scores of PTBP3 in the TMAs. The results revealed that high PTBP3 levels correlated with poor overall survival (p < 0.001, log rank test, Figure 1D) and disease-free survival (p < 0.001, log rank test, Figure 1E).

**Table 1:** Relationship between PTBP3 expression and clinicopathological features of patients with renal cancer

Variables	PTBP3 expression (n = 302 cases)		
	Low (%)	High (%)	*P*^a^
All patients	209 (100)	93 (100)	
Age (years)			0.053
≤56	108 (52)	38 (41)	
>56	101 (48)	55 (59)	
Gender			0.041
Males	135 (65)	65 (70)	
Females	74 (35)	28 (30)	
Depth of invasion			0.148
T1/T2	174 (65)	65 (70)	
T3/T4	35 (35)	28 (30)	
Lymph node metastasis			**0.046**
N0	196(94)	81 (87)	
N1/N2/N3	13 (6)	12 (13)	
Distant metastasis			**0.028**
M0	182 (87)	72 (77)	
M1	27 (13)	21 (23)	
TNM stage			**0.036**
I/II	160 (77)	60 (65)	
III/IV	49 (23)	33 (35)	
Tumor diameter			0.148
<7cm	174(83)	72 (77)	
≥7cm	35 (17)	21 (23)	

a Two -sided Fisher’s exact tests.

To further examine whether PTBP3 expression was an independent prognostic factor for RCC, we used univariate and multivariate Cox regression models to confirm the prognostic value of PTBP3 expression in RCC. Univariate Cox regression analysis suggested that PTBP3 expression was a significant prognostic factor for the overall ratio (HR 0.487, 95% confidence interval [CI], 0.361–0.656; p < 0.001) and disease-free survival (HR 0.486, 95%CI, 0.354–0.669; p < 0.001) in patients with RCC (Table S1). In multivariate Cox regression analysis, we also found that PTBP3 expression was an independent prognostic marker for both overall survival (HR 0.549, 95%CI, 0.401–0.751; p < 0.001) and disease-free survival (HR 0.524, 95%CI, 0.375–0.732; p < 0.001) in patients with RCC (Table S2). Therefore, we assessed the biological functions and molecular mechanisms of PTBP3 in RCC.

### Polypyrimidine tract-binding protein 3 promotes growth, migration, and invasion of renal cell carcinoma *in vivo*

**Figure 2:**
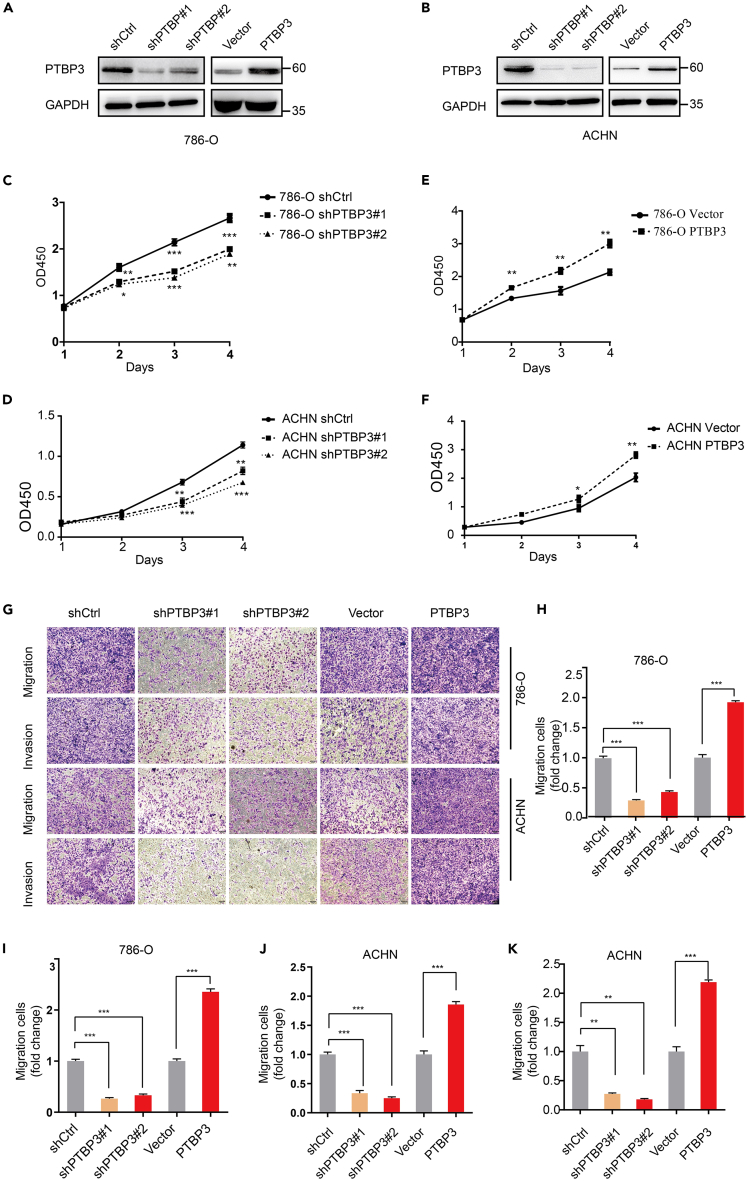
PTBP3 promotes growth, migration, and invasion of renal cell carcinoma *in vivo* (A and B) Detection of PTBP3 protein level in PTBP3 knockdown (KD) and over-expression (OE) 786-O and ACHN stable cell lines. GAPDH was used as a loading control. (C‒F) Effect of PTBP3 KD or OE on 786-O and ACHN cells proliferation as assessed by Cell Counting Kit-8 (CCK8) assays. ∗p < 0.05, ∗∗p < 0.01, ∗∗∗p < 0.001. (G‒K) Relative migration and invasion fold changes in 786-O and ACHN cells ± PTBP3 KD/OE. Statistical analysis was conducted using unpaired t test; Data are presented as the means ± SD. ∗p < 0.05, ∗∗p < 0.01, ∗∗∗p < 0.001.

As our RCC cohort showed that PTBP3 expression was associated with RCC progression, we investigated the biological function of PTBP3 in RCC. Firstly, we detected PTBP3 expression levels in different RCC cancer cell lines, data showed PTBP3 was higher expressed in 786-O, ACHN and Ketr3 RCC cell lines than normal kidney derived HK2 cell (Figure S1) To investigate the role of PTBP3 in RCC progression, 786-O and ACHN cells were stably infected with lentivirus-mediated PTBP3 shRNA and control shRNA or transfected with PTBP3 overexpression and control lentivirus (Figures 2A and 2B). To determine whether PTBP3 regulates the activity of renal cell carcinoma cells, we measured the viability of stable knockdown (KD) and overexpression (OE) of PTBP3 in 786-O and ACHN cells using cell counting kit-8 (CCK8). CCK8 assays showed that PTBP3 KD inhibited the proliferation of 786-O and ACHN cells (Figures 2C and 2D). In contrast, 786-O and ACHN cells overexpressing PTBP3 exhibited significantly higher proliferation (Figures 2E and 2F). To investigate whether PTBP3 contributes to RCC invasiveness of renal cell carcinoma, we tested the effect of PTBP3 on cell migration and invasion *in vitro*, which are two key features of the metastatic phenotype.^23^ Transwell assays showed that PTBP3 KD and OE inhibited and increased the migration and invasion abilities, respectively (Figures 2G–2K).

### Polypyrimidine tract-binding protein 3 binds to high-mobility group A1 mRNA

**Figure 3:**
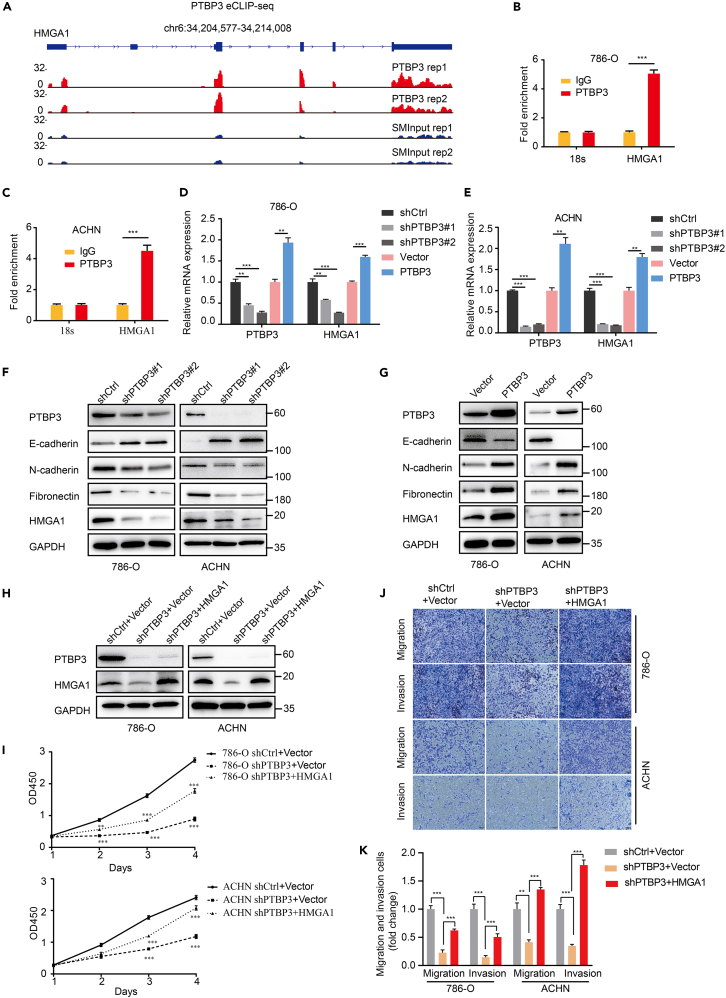
PTBP3 promotes HMGA1 expression and regulating renal cancer cells malignant through HMGA1 (A) The eCLIP-seq mapped PTBP3 binding events are shown on the HMGA1 transcript. (B and C) RNA immunoprecipitation with PTBP3 antibody in 786-O and ACHN cells, the co-immunoprecipitated HMGA1 RNA was detected by qRT-PCR, 18s RNA was used as a negative control, ∗∗∗p < 0.001. (D and E) HMGA1 mRNA expressions were analyzed in 786-O and ACHN cells ± PTBP3 KD/OE by qRT-PCR. The relative mRNA expression levels were normalized to GAPDH. ∗∗p < 0.01, ∗∗∗p < 0.001. (F and G) Western blots of EMT markers and HMGA1 in 786-O and ACHN cells ± PTBP3 KD/OE. GAPDH was used as a loading control. (H) 786-O and ACHN cells with PTBP3 KD were co-transfected with expression vectors encoding either empty vector, HMGA1 and assessed by Western blot for HMGA1 and PTBP3 expression. GAPDH was used as a loading control. (I) Effects of HMGA1 expression on the proliferation of 786-O and ACHN cells with PTBP3 KD by Cell Counting Kit-8 (CCK8) assays. ∗∗p < 0.01, ∗∗∗p < 0.001. (J and K) Effects of HMGA1 expression on the migration and invasion of 786-O and ACHN cells with PTBP3 KD. Statistical analysis was conducted using unpaired t test; Data are presented as the means ± SD. ∗∗p < 0.01, ∗∗∗p < 0.001.

To investigate the possible molecular mechanism by which PTBP3 regulates renal cancer malignancy, we analyzed eCLIP-SEQ data on genome-wide PTBP3-RNA interactions in HCT116 cells. We identified multiple sites of association between PTBP3 and HMGA, particularly in the 3′-UTR (Figure 3A). The interaction between PTBP3 and HMGA1 mRNA was subsequently confirmed in 786-O and ACHN cells (Figures 3B and 3C) by RNA immunoprecipitation. To determine the effect of PTBP3 on HMGA1 mRNA, we assessed the expression of HMGA1 in renal cancer cells with PTBP3 overexpression or knockdown. The mRNA levels of HMGA1 were determined by real-time quantitative PCR, which showed that PTBP3 knockdown significantly repressed HMGA1 mRNA expression, whereas PTBP3 overexpression significantly increased HMGA1 mRNA expression in 786-O and ACHN cells (Figures 3D and 3E).

### Polypyrimidine tract-binding protein 3 promotes HMGA1 expression and regulating renal cancer cells malignant through high-mobility group A1

HMGA1 plays an important role in tumor metastasis and EMT progress.^14^ We showed that the protein levels of HMGA1 were also downregulated or upregulated by PTBP3 knockdown or overexpression in 786-O and ACHN cells (Figures 3Fand 3G). In addition, the known EMT markers regulated by HMGA1 changed according to PTBP3 knockdown or overexpression; E-cadherin protein levels were upregulated and N-cadherin and Fibronectin were downregulated in 786-O and ACHN cells with PTBP3 knockdown (Figure 3F), E-cadherin protein levels were downregulated, and N-cadherin and Fibronectin were upregulated in 786-O and ACHN cells with PTBP3 overexpression (Figure 3G). Real-time PCR experiments also revealed the same mRNA expression changes in the EMT markers (Figure S2).

Our results demonstrate that PTBP3 promotes the growth and metastasis of renal cell carcinoma cells. Furthermore, we assessed the role of HMGA1 in PTBP3 induced migration and invasion of RCC cells. We ectopically expressed HMGA1 in the 786-O and ACHN PTBP3 KD cells (Figure 3H). The data showed that the ectopic expression of HMGA1 restored the growth, migration, and invasion capacity of PTBP3 KD cells (Figures 3I–3K), which further supports HMGA1 as a critical target of PTBP3.

### Knockdown of polypyrimidine tract-binding protein 3 decrease high-mobility group A1 mRNA stability

**Figure 4:**
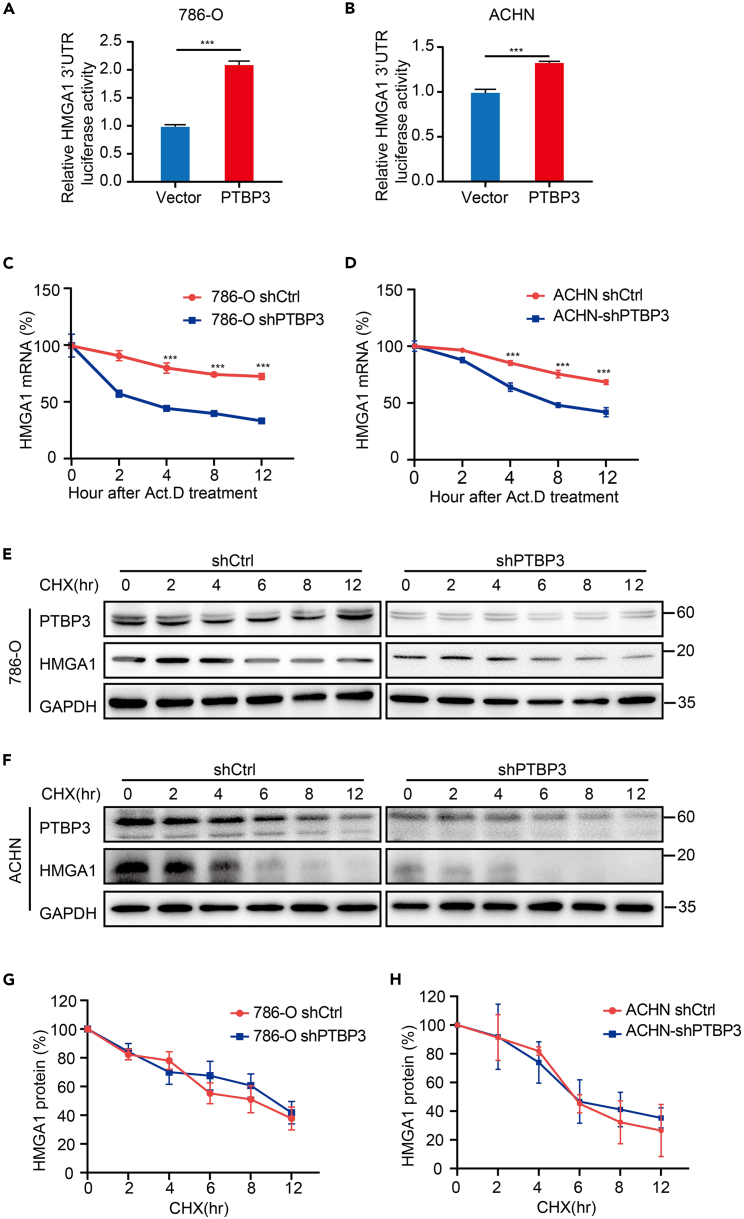
PTBP3 regulates HMGA1 mRNA stability and does not regulate HMGA1 protein stability (A and B) Effects of PTBP3 on HMGA1 3′UTR luciferase activity. PTBP3 and HMGA1 3′UTR Renilla Luciferase reporter plasmid were co-transfected to 786-O and ACHN cells, the relative luciferase activity was analyzed 24 h after transfection and normalized to Renilla activity. Data are presented as the means ± SD, n = 3. ∗∗∗p < 0.001. (C and D) Relative HMGA1 mRNA remaining were calculated versus the time of incubation with 10 μg/mL actinomycin D (Act. D) by qRT-PCR. Data are presented as the means ± SD, n = 3. ∗∗∗p < 0.001. (E and F) Western blot showing HMGA1 protein levels in 786-O and ACHN cells ± PTBP3 KD at the indicated time points after cyclohexamide (CHX). GAPDH was used a loading control. (G and H) Graphical representation of HMGA1 protein levels from (E and F) based on densitometry and normalized to GAPDH. Statistical analysis was conducted using unpaired t test; Data are presented as the means ± SD.

It has been reported that PTBP3 regulates mRNA turnover through binding to mRNA 3′UTR transcripts.^8^^,^^10^ Our data showed that PTBP3 binds to HMGA1 mRNA 3′-UTR and passively regulates HMGA1 mRNA levels (Figure 3). We hypothesized that PTBP3 stabilizes HMGA1 mRNA expression. To verify this, we first used dual-luciferase reporter assays to determine the role of PTBP3 in the regulation of HMGA1 stability. HMGA1 3′UTR cDNA was cloned into the 3′UTR of luciferase cDNA to simulate the regulation of mRNA stability, and it was found that PTBP3 overexpression enhanced luciferase activity in 786-O and ACHN cells (Figures 4A and 4B), suggesting that PTBP3 may stabilize HMGA1 mRNA through HMGA1 3′UTR. Actinomycin D (Act. D) was used to block transcription in shCtrl and shPTBP3 cells, and the half-life of HMGA1 mRNA was assessed. Our data demonstrated that PTBP3 knockdown decreased the half-life of HMGA1 mRNA in 786-O and ACHN cells (Figures 4C and 4D). We used cycloheximide (CHX) to block protein synthesis and measured HMGA1 decay curves. We measured HMGA1 protein levels at different time points and found that PTBP3 knockdown had little effect on HMGA1 protein half-life (Figures 4E–4H). These data strongly suggested that PTBP3 increased HMGA1 mRNA levels by enhancing HMGA1 mRNA stability.

### Polypyrimidine tract-binding protein 3/ insulin-like growth factor 2 mRNA-binding proteins blocks let-7 mediated high-mobility group A1 mRNA degradation

**Figure 5:**
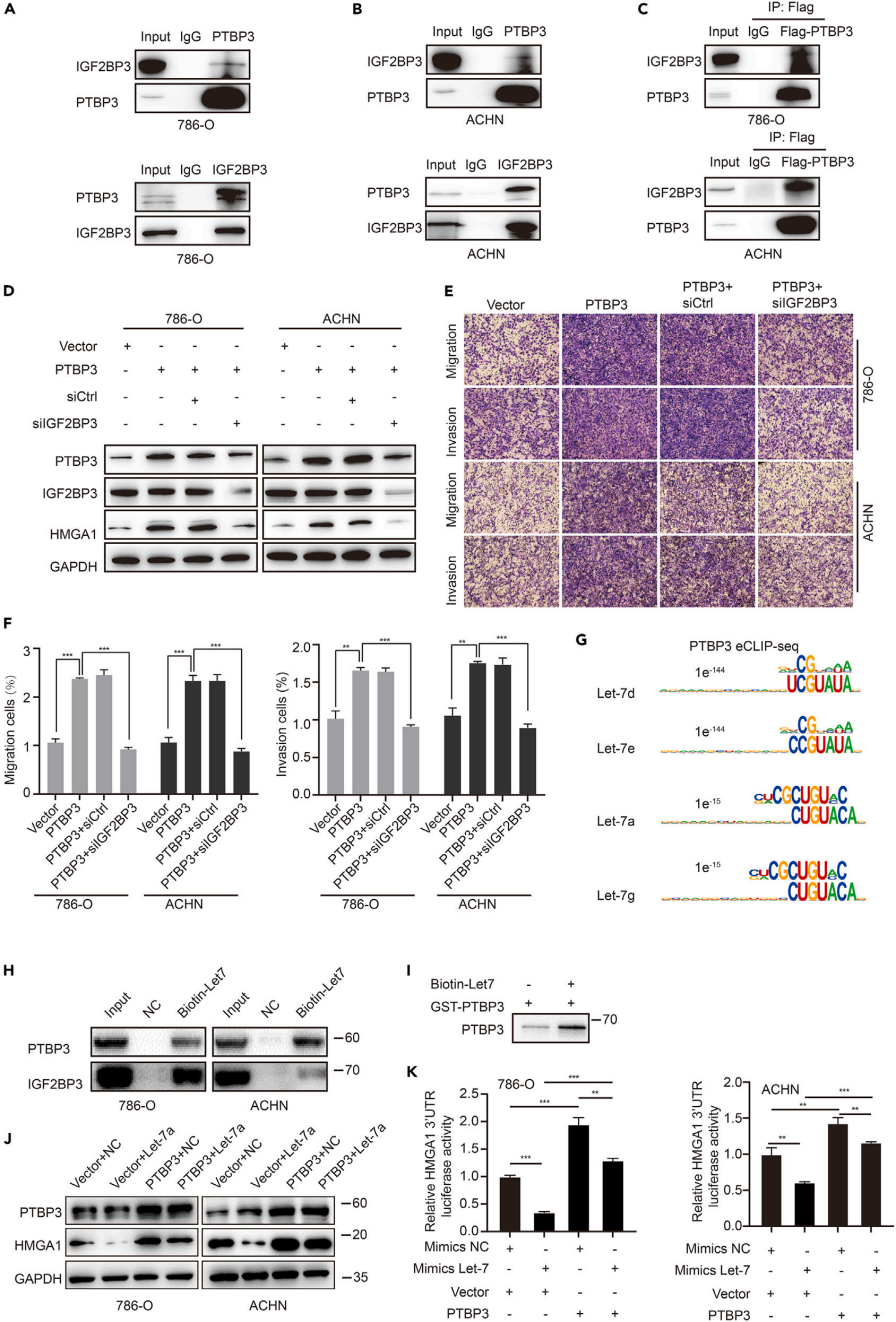
PTBP3 interacts with IGF2BP3 and prevents let-7 mediated HMGA1 silencing (A and B) Immunoprecipitation with anti-PTBP3 or anti-IGF2BP3 antibody in 786-O and ACHN cells, anti-IgG antibody as a negative control. (C) Immunoprecipitation with anti-Flag antibody and anti-IgG antibody as a negative control in RCC cells. (D) Effect of IGF2BP3 KD on the expression of HMGA1 protein level, as assessed by Western blot. (E and F) Effects of IGF2BP3 KD in cell migration and invasion (G) HOMER motif analysis results showed several binding consensus sequences of PTBP3 were similar to the let-7 miRNAs, PTBP3 eCLIP-seq data was analyzed. (H) RNA pull down assays were used to test the interactions between PTBP3 and let-7. Biotin labeled let-7a was used to perform RNA pull down assays using 786-O and ACHN cell lysates and western blots were performed to test PTBP3 and IGF2BP3, IGF2BP3 was used as a positive control. (I) Western blot of PTBP3 using RNA pull down samples by biotin labeled let-7a and PTBP3 GST fusion protein. (J) Western blots of HMGA1 in PTBP3 and let-7a co-transfected 786-O and ACHN cells. PTBP3 plasmids and let-7a mimics were co-transfected to 786-O and ACHN cells, cells were harvested at 24 h after transfection and western blots were performed to calculate HMGA1 and PTBP3 expressions, GAPDH was used as a loading control. (K) Effects of PTBP3 on let-7a mediated HMGA1 3′UTR luciferase activity silencing. PTBP3 plasmid and let-7a minics and HMGA1 3′UTR reporter plasmid were co-transfected to 786 and ACHN cells, the relative luciferase activities were analyzed 24 h after transfection. Statistical analysis was conducted using unpaired t test; Data are presented as the means ± SD, n = 3. ∗∗p < 0.01, ∗∗∗p < 0.001.

Previous studies have shown that IGF2BPs can interact with let-7 miRNAs and prevent let-7 target silencing.^20^^,^^21^^,^^22^ Therefore, we aimed to determine whether PTBP3 regulates HMGA1 by interacting with IGF2BP3 and let-7 miRNAs. The interaction between PTBP3 and IGF2BP3 was determined by co-immunoprecipitation (Co-IP). Co-IP results clearly indicated that IGF2BP3 interacts with PTBP3 in 786-O and ACHN cells (Figures 5A and 4B). Next, using PTBP3 FLAG tag pull-down followed by Co-IP, the data suggested that IGF2BP3 were associated with PTBP3 in 786-O and ACHN cells (Figure 5C). Then, we downregulated IGF2BP3 in PTBP3 OE 786-O and ACHN cells using IGF2BP3 siRNA, we noticed that IGF2BP3 KD can partially decrease HMGA1 expression (Figure 5D), as well as repressing cell migration and invasion induced by PTBP3 overexpression (Figures 5E and 5F). We also repressed IGF2BP3 in PTBP3 KD 786-O and ACHN cells, data showed siIGF2BP3 could further downregulate HMGA1 expression, we also noticed a repression of PTBP3 when silencing IGF2BP3 (Figure S3), it seems IGF2BP3 may be a regulator of PTBP3. These results provided remarkable evidence that PTBP3 and IGF2BP3 may form a complex to regulate HMGA1 transcription in renal cancer.

We further investigated the interactions between PTBP3 and let-7. We checked the PTBP3 eCLIP-seq data, and the HOMER motif analysis results showed that several binding consensus sequences of PTBP3 were similar to those of let-7 miRNAs (Figure 5G), suggesting a possible interaction between PTBP3 and let-7 miRNAs. To test this, we used RNA pull-down assays to verify the interaction between PTBP3 and let-7 miRNAs, and the results showed that let-7a RNA Oligos interacted with endogenous PTBP3 protein (Figure 5H). Furthermore, let-7a directly interacted with purified GST-PTBP3 protein (Figure 5I), suggesting a direct interaction between PTBP3 and let-7 miRNAs. We co-transfected PTBP3 and let-7a to 786-O and ACHN cells, western blot assays showed that PTBP3 could partly restore let-7a induced HMGA1 repression (Figure 5J). Dual-luciferase reporter assays were used to investigate the effects of PTBP3 on let-7 mediated HMGA1 3′UTR activity. It was found that let-7a repressed HMGA1 3′UTR luciferase activity, whereas PTBP3 overexpression restored the luciferase activity repression caused by let-7a (Figure 5K). These results suggested that PTBP3 interacts with let-7 miRNAs and prevents let-7 miRNA-mediated HMGA1 silencing.

### Polypyrimidine tract-binding protein 3 promoted renal cancer growth and metastasis through high-mobility group A1

**Figure 6:**
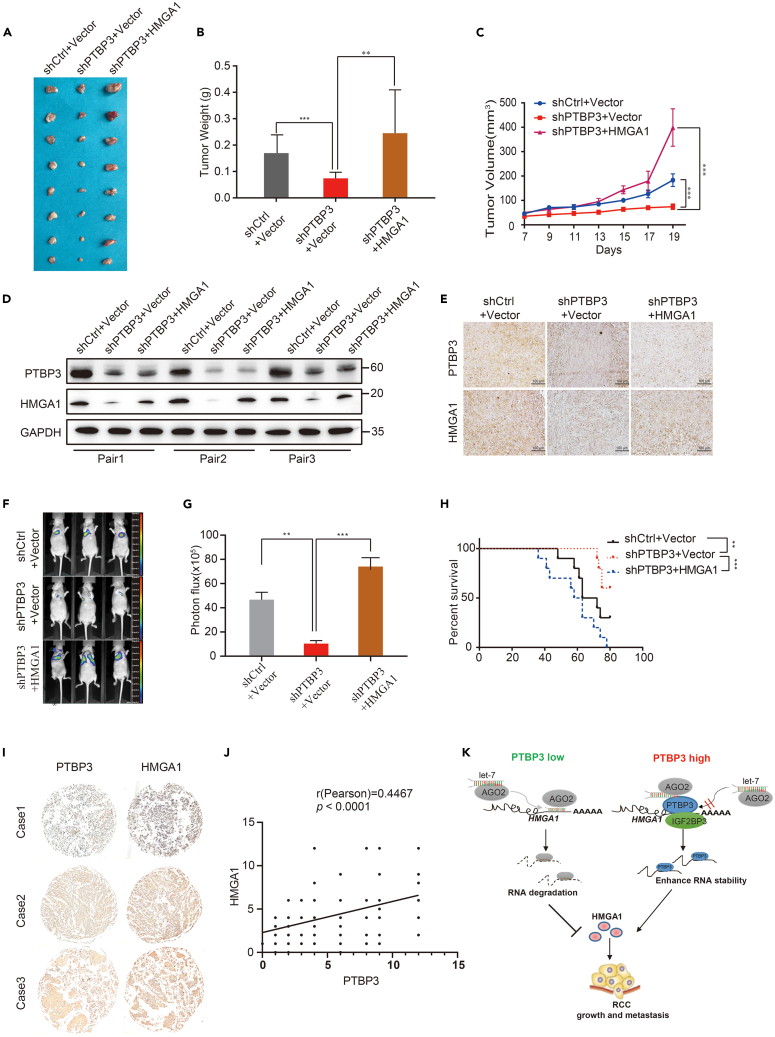
PTBP3 promoted renal cancer growth and metastasis *in vivo* (A‒C) Effect of PTBP3 KD and PTBP3 KD with overexpression HMGA1 in 786-O cells on the xenograft model was assessed by evaluating tumor volume and tumor weight. 5×10^6^ 786-O PTBP3 Con/KD cells and PTBP3 KD with overexpression HMGA1 and Matrigel (Corning; 1:1 ratio) were subcutaneously injected into the abdominal flanks of each mice, respectively.∗∗∗p < 0.001. (D) Western blot of HMGA1 expression in tumor xenografts formed. GAPDH was used as a loading control. (E) The tumor sections were performed immunochemistry staining by antibody against PTBP3 and HMGA1, representative images were shown. (F) Representative bioluminescence images of lung metastases in mice via tail vein injection of indicated cells. (G) The metastases were quantified by measuring the photo flux. Data are presented as the mean ± standard deviation,∗∗p < 0.01,∗∗∗p < 0.001. (H) Kaplan-Meier survival curves depicting overall survival of the mice with the injection of 786-O PTBP3 Con/KD cells and PTBP3 KD with overexpression HMGA1, n = 10. (I and J) Analysis of the correlation coefficients between PTBP3 and HMGA1 in 201 RCC tissue samples. The correlation coefficients were calculated by Pearson test, p < 0.0001, representative images were shown. (K) A cartoon summarizing our findings. PTBP3 prevents let-7 mediated HMGA1 mRNA degradation in RCC, thereby promoting RCC growth, and metastasis.

To observe the effect of xenografts on tumor growth *in vivo*, we used a nude mouse model to evaluate the effect of 786-O PTBP3 KD cells and HMGA1 overexpression in 786-O PTBP3 KD cells on tumor growth. Compared to the control group, mice with xenografts of PTBP3 KD tumor cells formed smaller tumors in terms of tumor size and weight *in vivo* (Figures 6A and 6B). Re-expression of HMGA1 completely rescued the growth capacity of PTBP3 KD *in vivo* (Figure 6C). Western blotting and IHC staining of tissue sections from nude mice were performed to evaluate PTBP3 and HMGA1 expression (Figures 6D, 6E, and S4). These results suggested that PTBP3 promotes RCC cell growth via HMGA1.

In the present study, PTBP3 promoted HMGA1 expression and renal cancer cell migration and invasion. It is well known that metastasis and invasion are essential for cancer metastasis,^24^ we investigated the role of PTBP3 in RCC metastasis *in vivo*. 786-*O*-Luc control, 786-*O*-Luc PTBP3 KD, and HMGA1 overexpression in 786-*O*-Luc PTBP3 KD cells were injected separately into the tail veins of nude mice. Interestingly, 786-*O*-Luc control and HMGA1 overexpression in 786-*O*-Luc PTBP3 KD cells significantly enhanced lung metastasis in nude mice, whereas 786-*O*-Luc-PTBP3 KD cells metastasized to the lungs of nude mice, as confirmed by bioluminescence imaging (Figures 6F and 6G). The survival of mice was also determined. PTBP3 KD increased survival rates, whereas the re-expression of HMGA1 decreased survival (Figure 6H). In addition, in 201 RCC tissue samples, the relative protein expression levels of PTBP3 were concordant to HMGA1 (Figures 6I and 6J).Therefore, HMGA1 may be responsible for the metastasis of RCC induced by PTBP3.

In conclusion, our study reveals an important posttranscriptional mechanism of PTBP3. Our results suggested a model in which PTBP3 prevents let-7 miRNA-mediated HMGA1 silencing during RCC progression. We showed PTBP3 is an important regulator of HMGA1 and promotes RCC growth and metastasis (Figure 6K).

## Discussion

The PTB family is comprised of three members: PTBP1, PTBP2, and PTBP3. RBPs bind to RNA directly through one or more RNA-binding domains, including RNA recognition motifs, K-homology domains, and zinc fingers.^25^^,^^26^ RBPs regulate RNA expression by affecting various aspects of gene metabolism.^27^ The functions and mechanisms of PTBP1 and PTBP2 have been studied extensively. PTBP1 and PTBP2 interact with RNAs to regulate splicing, stabilization, localization, and translation.^28^ As an important member of the PTB family, PTBP3 has been reported to be overexpressed and play a role in the progression of various cancers.^8^^,^^9^^,^^11^^,^^12^ However, the function and mechanism of action of PTBP3 in renal cell carcinoma remains unclear.

In this study, we explored the biological function of PTBP3 in RCC progression, and investigated its potential molecular mechanisms. Our results showed that PTBP3 expression was increased in human RCC, and high PTBP3 expression was correlated with clinicopathological parameters such as lymph node metastasis, distant metastasis, TNM stage and poor five-year overall survival, and disease-free survival. In addition, univariate and multivariate Cox regression analyses showed that high PTBP3 expression was an independent adverse prognostic factor in patients with RCC. These results suggest that PTBP3 plays a significant role in RCC progression and could function as a potential clinical prognostic predictor in patients with RCC.

The HMGAs protein has been reported to be overexpressed in various human cancers, including epithelial cancers such as breast cancer,^29^ colorectal cancer,^30^^,^^31^ lung cancer^32^^,^^33^ uterine cancer,^34^ and mesenchymal tumors such as glioma/glioblastoma,^35^ lipoma/liposarcoma,^36^ leiomyoma,^37^ fibroma/fibrosarcoma^38^ and osteosarcoma,^39^ and is often associated with metastases and a poor prognosis.^14^ HMGA1 and HMGA2 are two important members of it and play an important role in tumor progression.^14^^,^^40^ Our data demonstrated that PTBP3 promotes the proliferation of renal cell carcinoma cells. Additionally, PTBP3 promotes the migration and invasion abilities of renal cancer cells by regulating HMGA1 expression *in vivo*. HMGA1 overexpression restored the effect of PTBP3 downregulation on the proliferation, migration, and invasion of renal cell carcinoma *in vivo*. In addition, PTBP3 promotes the proliferation and pulmonary metastasis of renal cancer cells in an animal xenograft model. Similarly, HMGA1 overexpression restored the effect of PTBP3 downregulation on proliferation and pulmonary metastasis of renal cell carcinoma cells *in vitro*. The survival time of mice in the HMGA1 high expression group was significantly prolonged. These results suggested that we explore the potential mechanism by which PTBP3 regulates HMGA1 expression and activity.

HMGA1 proteins do not have transcriptional activity; however, by interacting with transcriptional mechanisms, they alter chromatin structure, thereby negatively or positively regulating the transcriptional activity of several genes.^41^^,^^42^ HMGA1 acts as an oncogene through transcriptional regulation and protein-protein interactions.^43^ For example, HMGA1 protein expression indicates adverse clinical outcomes in breast cancer.^29^ HMGA1 is also a target gene for let-7 miRNAs.^44^^,^^45^ Several studies have shown that IGF2BPs can bind to let-7 miRNAs and blocked Let-7 target genes, such as HMGA1 and HMGA2.^20^^,^^21^^,^^22^ Enhanced CLIP (eCLIP) is critical for revealing the binding profiles of RBPs, and eCLIP-seq data showed the binding of PTBP3 to HMGA1 3′-UTR and let-7 transcripts. Moreover, PTBP3 prevents let-7 mediated HMGA1 silencing in RCC and promotes RCC growth and metastasis through HMGA1. We previously showed that PTBP3 interacts with AGO2 and prevents the miRNA-mediated degradation of ZEB1 mRNA.^8^ These results suggested that PTBP3 regulates target gene expression by forming complexes with mRNAs, miRNAs, and RBPs.

Insulin-like growth factor 2 (IGF2) mRNA-binding protein 3 (IMP3, IGF2BP3) is a member of the RNA binding protein family (IMP1, IMP2, and IMP3) involved in RNA localization, translation, and stability.^46^ In this work, our data showed that PTBP3 protein and IGF2BP3 protein could bind to each other, both in exogenous or endogenous. IGF2BP3 silencing decreased HMGA1 protein levels accordingly in PTBP3 overexpressing renal cell carcinoma cells. Hence, in our study, we demonstrated that PTBP3 interact with IGF2BP3 to regulate the stability of HMGA1 mRNA by acting on the 3′UTR region of HMGA1. We also found a downregulation of PTBP3 when the silencing of IGF2BP3 (Figures 5D and S3), it suggested a potential role of IGF2BP3 in regulating PTBP3 expression, it would be further investigated in the future. IGF2BPs worked as m^6^A readers and regulating mRNA stability and translation.^47^ Considering that the RNA binding protein PTBP3 interacted with IGF2BP3 and regulated mRNA stability, it suggested that PTBP3 might be involved in the m^6^A regulating pathway, but it needs further studies in this area.

In summary, this study provides new evidence of the clinical and biological significance of PTBP3 in RCC. Our data showed that PTBP3 expression was increased in human RCC, and high PTBP3 expression was correlated with poor five-year overall survival and disease-free survival. Furthermore, PTBP3 promotes renal cancer cell proliferation, migration, and invasion *in vitro* as well as tumor growth and metastasis *in vivo*. Importantly, we discovered that PTBP3/IGF2BP3 prevents let-7 miRNA-mediated HMGA1 silencing during RCC progression. Our data showed that PTBP3 is an important regulator of HMGA1 and promotes RCC growth and metastasis. These findings shed light on the potential of PTBP3 as a biomarker and target for RCC prognosis and therapy.

### Limitations of the study

The protein level of PTBP3 was upregulated in RCC tissues, while the mRNA level of PTBP3 was downregulated in RCC tissues. It seems PTBP3 may be regulated at a post-transcriptional level, but we did not study the mechanisms of the upregulation of PTBP3 in RCC. Our study also showed a regulation of IGF2BP3 in PTBP3 expression, but the mechanisms were unclear, further research is needed to fully understand the molecular mechanism by which IGF2BP3 regulates PTBP3 expression.

## STAR★Methods

### Key resources table

**Table:** 

REAGENT or RESOURCE	SOURCE	IDENTIFIER
**Antibodies**		

ROD1(PTBP3) antibody	Santa Cruz	Cat# sc-100845; RRID:AB_2182326
ROD1 antibody	Proteintech	Cat#14027-1-AP RRID:AB_2182321
GAPDH antibody	Santa Cruz	Cat# sc-32233; RRID:AB_627679
E-cadherin antibody	BD Biosciences	Cat# 610181; RRID:AB_397580
N-cadherin antibody	BD Biosciences	Cat# 610920; RRID:AB_2077527
Fibronectin antibody	BD Biosciences	Cat# 610077; RRID:AB_2105706
HMGA1 antibody	ABclonal	Cat# A1635; RRID:AB_2763693
IGF2BP3 antibody	Proteintech	Cat# 14642-1-AP; RRID:AB_2122782
Secondary antibodies	Proteintech	Cat# SA00001-2; RRID:AB_2722564 Cat# SA00001-1; RRID:AB_2722565
VeriBlot for IP Detection	Abcam	Cat# ab131366 RRID:AB_2892718

**Bacterial and virus strains**		

TransStbl3 Chemically Competent Cell	Transgen	Cat# CD521

**Biological samples**		

Paired of Renal cancer tissue microarray slides	Shanghai Outdo Biotech Company	N/A
Renal cancer tissue microarray slides	the Affiliated Hospitals of Xuzhou Medical University	N/A

**Chemicals, peptides, and recombinant proteins**		

Cycloheximide	MedChemExpress	Cat# HY-12320
Actinomycin D	MedChemExpress	Cat# HY-17559
Puromycin	Beyotime	Cat# ST551
ROD1 fusion protein	Proteintech	Cat# Ag5172

**Critical commercial assays**		

HiScript III 1st Strand cDNA Synthesis Kit	Vazyme	Cat# R312-01
UltraSYBR One Step RT-qPCR Kit	CWBIO	Cat# CW0659
Cell Counting Kit-8	KeyGEN BioTECH	Cat# KGA317s
siLentFect Lipid Reagent	Bio-Rad	Cat# 1703360
Dual-Luciferase® Reporter Assay System	Promega	Cat# E1910

**Deposited data**		

Mendeley Data: https://doi.org/10.17632/3b7gvbbf5m.1		

**Experimental models: Cell lines**		

786-O	China Center for Type Culture Collection	N/A
ACHN	China Center for Type Culture Collection	N/A
Ketr3	China Center for Type Culture Collection	N/A
OSRC2	China Center for Type Culture Collection	N/A
HK-2	China Center for Type Culture Collection	N/A
HEK-293T	China Center for Type Culture Collection	N/A

**Experimental models: Organisms/strains**		

Mouse: BALB/c Nude	Vital River	N/A

**Oligonucleotides**		

Primers for qPCR, siRNA and shRNA sequences, refer to Tables S3 and S4		

**Recombinant DNA**		

pCDH-CMV-MCS-EF1-GreenPuro	System Biosciences	Cat# CD513B-1
pCDH-CMV-PTBP3-EF1-GreenPuro	This paper	N/A
PLKO.1 vector	Addgene	Cat# 10878
psPAX2	Addgene	Cat# 12260
pMD2.G	Addgene	Cat# 12259
pLV-HMGA1-Neomycin	This paper	N/A
psiCHECK2-HMGA1-3’UTR	This paper	N/A

**Software and algorithms**		

ImageJ	National Institutes of Health	https://imagej.net/
GraphPad Prism 8 software	GraphPad Prism Software Inc	https://www.graphpad.com/
Image pro	Media Cybernetics	https://mediacy.com/image-pro/

### Resource availability

#### Lead contact

The relevant experimental reagents, experimental methods, and related data of this study can be obtained by contacting Pingfu Hou (houpingfu@163.com).

#### Materials availability

The study did not generate new unique reagents.

#### Data and code availability

All data reported in this paper will be shared by the lead contact upon request.

This paper does not report the original code.

Any additional information required to reanalyze the data reported in this paper is available from the lead contact upon request.

### Experimental model and study participant details

#### Clinical samples

Tissue microarray (TMA) slides, which included the examination of 74 pairs of renal cancer tissues, were purchased from the Shanghai Outdo Biotech Company and included 74 patients who underwent radical nephrectomy from 2006 to 2008. TMAs, including 302 RCC tissue samples, were obtained from the Affiliated Hospital of Xuzhou Medical University from 2005 to 2008 in China. Clinical and pathological information was obtained from the medical records of the Affiliated Hospitals of Xuzhou Medical University. All the participants provided informed consent. Ethical approval for human subjects was obtained from the Ethics Committee of the Affiliated Hospital of Xuzhou Medical University (XYFY2022-KL022-01). The clinical data are shown in Tables S3 and S4. For immunohistochemistry (IHC), tissues were stained overnight at 4C with anti-PTBP3 (sc-100845, Santa Cruz) antibody was applied at a 1:100 dilution and anti-HMGA1 (A1635, ABclonal, Wuhan, China) antibody at a 1:100 dilution.

#### *In vivo* experiments

The female BALB/c nude mice (6–8 weeks old) were purchased from Beijing Vital River Laboratory Animal Technology Co., Ltd. (Beijing, China). ALL animal experiments were approved by the Animal Care and Use Committee of Xuzhou Medical University (NO.202306T013). The mice were maintained in a controlled environment with controlled temperature (∼25°C), humidity (50–70%) and (light, 07:00; dark, 22:00). The water and mouse feed were sterilised by uperisation and were freely available. Briefly, the stable cell lines of 786-O-shCtrl-Vector\shPTBP3-Vector\shPTBP3-HMGA1 were established. Cells were injected subcutaneously (5×10^6^) or via the caudal vein (1×10^6^) into Female BALB/c nude mice (8 weeks old). Tumor volume was calculated using the formula V = a × (b × b)/2, where a is the largest and b is the smallest diameter. For bioluminescence imaging, mice were injected with 100 mg/kg D-luciferin before imaging. After anesthesia by Ketamine, images were captured using the Night OWL II LB983 (Berthold Technologies) and the intensity of bioluminescence was measured for calculating the lung metastasis. Finally, CO_2_ were used for euthanasia of the mice.

### Method details

#### Cell lines and cell culture conditions

The RCC cell lines were obtained from the Cell Bank of the Chinese Academy of Sciences. 786-O cell lines were cultured in RPMI 1640 medium supplemented with 10% FBS, ACHN cells were cultured in DMEM medium supplemented with 10% FBS, and incubated in at 37°C humidified incubator with 5% CO_2_. The cell lines were authenticated by short tandem repeat (STR) profiling by Genewiz, CN. All experiments were performed with mycoplasma-free cells.

#### Western blot and antibodies

The total protein of cells were harvested using RIPA lysis buffer for 30 min on ice. The cell suspension was collected by centrifuging in a microcentrifuge for 10 min at 4°C, 12,000rpm.The concentration of proteins lysis was calculated by BCA method. Proteins were loaded and running in the SDS-PAGE gel, and then transferred the protein from the gel to the membrane. Block the membrane for 1 h at room temperature using 5% skim milk, then incubate the membrane with appropriate dilutions of primary antibody in blocking buffer overnight 4°C, and following with washing membrane for three times by TBST, and incubate the membrane with secondary antibody in blocking buffer at room temperature for 1 h. At last, images were taken by Tanon 5200 for chemiluminescence. Antibody against PTBP3 (sc-100845, Santa Cruz), GAPDH (sc-32233, Santa Cruz), E-cadherin (610181, BD Biosciences), N-cadherin (610920, BD Biosciences), Fibronectin (610077, BD Biosciences), HMGA1 (A1635, ABclonal, Wuhan, China), IGF2BP3 (14642-1-AP, Proteintech Group) were used for Western blot assays.

#### Transient transfections

Let-7a mimics and siRNAs were purchased from GenePharma (Suzhou GenePharma Co., Ltd.) and transfected with siLentFect Lipid Reagent (Bio-Rad Laboratories, Inc.) according to the manufacturer's protocol when RCC cells were grown to approximately 40%-50% confluency. Six hours after transfection, fresh medium was used instead of medium containing the transfection reagent. The siRNAs sequences were listed in Table S3.

#### Establishment of stable cell lines

The pCDH-CMV-PTBP3-EF1-GreenPuro or pLKO.1-shRNAs or pLV-HMGA1 vectors were co-transfected with psPAX2 and pMD2.G into HECK-293T cells, the lentivirus were collected 48 hours after transfection. Cells were incubated with lentivirus for 12 hours and then incubated with fresh medium. Puromycin or Geneticin were added at 48 hours after lentivirus infection. Stable cell lines were established after selecting using Puromycin or Geneticin for about two weeks. The PTBP3 shRNA sequences were listed in Table S5.

#### RNA extract, reverse transcription-PCR and qRT-PCR

RNA was extracted using TRIZOL (Invitrogen) and cDNA was synthesized using the HiScript 1st Strand cDNA Synthesis Kit (Vazyme Biotech, Nanjing, China). Real-time PCR was performed on an ABI-7500 instrument using the UltraSYBR One Step RT-qPCR Kit (CWBIO, Beijing, China). The primers used for the qRT-PCR analysis were listed in Table S6.

#### Cell migration, invasion assays and cellular proliferation

Cell migration and invasion assays were performed using 24-well inserts transwell chambers (8.0 μm, Corning). 5×10^4^ cells were added to the top chambers coated with or without Matrigel (BD Biosciences). Complete medium was added to the bottom wells and incubated 24–48 h, then cells adhered to the lower surface of the membrane were stained with 0.1% Crystal Violet and counted. Cell proliferation was calculated by CCK-8 kit according to the Cell Counting Kit-8 manufacturer's protocol (Jiangsu KeyGEN BioTECH Corp., Ltd.).

#### RNA immunoprecipitation and RNA pull-down

For RNA immunoprecipitation (RIP) assays, briefly, 2×10^7^ 786-O or ACHN cells were dissolved in RIP lysis buffer. Cell extracts were pre-defined with 50μl protein A/G-agarose at 4°C for 2 h. 3ug anti-PTBP3 or IgG antibody was added to the supernatant and incubated gently at 4°C for overnight. Then 50μl of protein A/G-agarose was added to the supernatant and incubated for 2 h at 4°C. Then, beads were cleaned and RNA was extracted by TRIZOL. At last, qRT-PCR was performed to calculate the interaction between PTBP3 protein and HMGA1 mRNA. For RNA pull-down assay, the Biotin-labeled let-7a and the negative control were synthesized by Suzhou GenePharma Co. Ltd. The PTBP3 fusion protein was obtained from Proteintech Group (Ag5172).

#### RNA and protein decay analysis

RNA stability was measured by incubating cells with 10 μg/ml actinomycin D (Act. D) and calculating the relative HMGA1 mRNA levels at different time points using qRT-PCR. RNA levels were normalized to GAPDH. Protein stability was measured by treating cells with 5 μg/ml cycloheximide(CHX) and calculating HMGA1 protein levels at different time points by western blotting. GAPDH was used as a loading control. Actinomycin D and cycloheximide were purchased from MedChem Express (Princeton, MA).

#### Luciferase assays

The HMGA1-3’UTR cDNA sequences was synthesized by GENEWIZ Co. Ltd and inserted into the luciferase reporter vector psiCHECK2 (Promega). A Dual-Luciferase® Reporter Assay System (Promega) was used to measure firefly and renilla luciferase activities according to the manufacturer’s protocol.

### Quantification and statistical analysis

GraphPad Prism 8 were used for statistical analyses. The chi-square test was used to evaluate the correlation between PTBP3 staining and clinicopathological parameters of patients with renal cancer. The Kaplan-Meier method and log-rank test were used to evaluate the correlation between PTBP3 expression and the survival time of renal cell carcinoma patients with RCC. Unpaired t-tests were used to determine the statistical significance of the intergroup differences. Data are shown as mean ±SD. Statistical significance was set at P <0.05.

## Supplemental information

Document S1. Figures S1‒S4

Document S2. Tables S1‒S6
