# Supplementary material for: Polypyrimidine tract-binding protein 3/insulin-like growth factor 2 mRNA-binding proteins 3/high-mobility group A1 axis promotes renal cancer growth and metastasis
Source: iScience. 2024 Feb 9;27(3):109158. doi: 10.1016/j.isci.2024.109158 (PMC10884747; doi:10.1016/j.isci.2024.109158)
Supplement: Document S1. Figures S1‒S4 [file mmc1.pdf]

iScience, Volume *27*

Supplemental information

Polypyrimidine tract-binding protein 3/insulin-like growth factor 2 mRNA-binding proteins 3/high-mobility group A1 axis promotes renal cancer growth and metastasis

Qianqing Wang, Fang Chen, Yu He, Yue Gao, Jiawen Wang, Sufang Chu, Pei Xie, Jiateng Zhong, Haixia Shan, Jin Bai, and Pingfu Hou

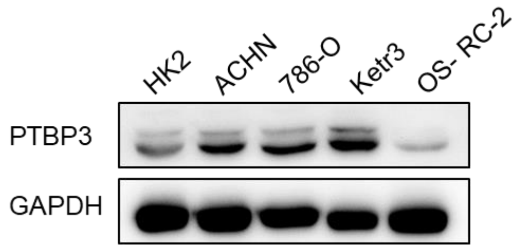

Figure S1. Protein expressions of PTBP3 in RCC cell lines. Detection of PTBP3 in different RCC cell lines by Western blots.

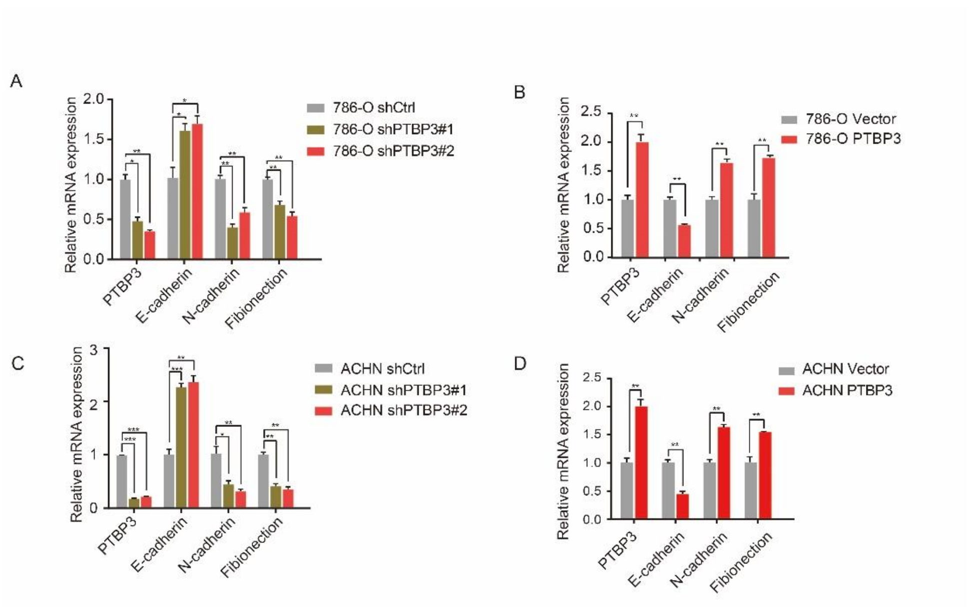

Figure S2: **Effects of PTBP3 KD/OE on the mRNA expression of EMT markers.** (A-D) Relative mRNA expression levels of PTBP3 and EMT markers in 786-O and ACHN cells ± PTBP3 KD/OE. The relative mRNA expression levels were normalized to GAPDH. Data are presented as the means ± SD, $*p<0.05$, $**p<0.01$, $***p<0.001$.

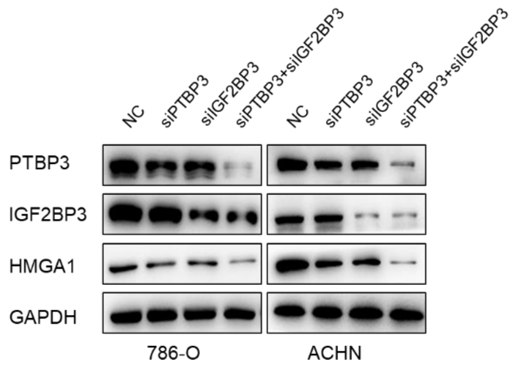

Figure S3. Western blots detection of HMGA1 in after silencing of PTBP3 or IGF2BP3 by siRNAs in 786-O and ACHN cells.

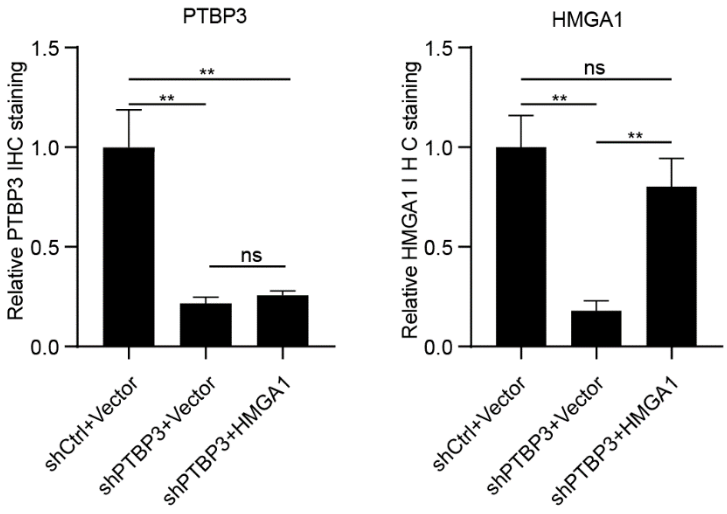

Figure S4. Relative IHC staining of PTBP3 and HMGA1 analyzed by Image-pro software.

Data are presented as the means ± SD, $^{**}p<0.01$.
