# Supplementary material for: Polypyrimidine tract-binding protein 3/insulin-like growth factor 2 mRNA-binding proteins 3/high-mobility group A1 axis promotes renal cancer growth and metastasis
Source: iScience. 2024 Feb 9;27(3):109158. doi: 10.1016/j.isci.2024.109158 (PMC10884747; doi:10.1016/j.isci.2024.109158)
Supplement: Document S2. Tables S1‒S6 [file mmc2.pdf]

Table S1. Univariate Cox regression analysis of PTBP3 expression and clinicopathologic variables predicting the survival of renal cancer patients

Variables*	Overall survival		Disease-specific survival	
	HR (95%CI)	*P*	HR (95%CI)	*P*
PTBP3	0.487 (0.361–0.656)	<0.001	0.486 (0.354–0.669)	<0.001
Age	0.923 (0.699–1.218)	0.570	0.934(0.697–1.252)	0.649
Gender	0.824 (0.618–1.097)	0.185	0.836 (0.617–1.131)	0.246
TNM stage	0.508 (0.374–0.690)	<0.001	0.604 (0.431–0.848)	0.004
LNM	0.188 (0.102–0.348)	<0.001	0.396 (0.098–1.606)	0.195
Tumor diameter	0.615 (0.438–0.863)	0.005	0.666 (0.440–0.964)	0.031
Depth of invasion	0.594 (0.422–0.837)	0.003	0.633 (0.437–0.918)	0.016

Abbreviations: HR: hazard ratio; CI: confidence interval; LNM: lymph node metastasis

*: PTBP3: low *vs* high; Age: ≤56 *vs* >56; Gender: male *vs* female; LNM: N0 *vs* N1, N2, N3; Depth of invasion: T1-T2 *vs* T3-T4; TNM stage was ranked as I-II *vs* III- IV; Tumor diameter: < 7cm *vs* ≥7cm.

Table S2. Multvariate Cox regression analysis on 5-year overall and disease specific survival of renal cancer patients

Variable*	Overall survival			Disease-specific survival		
	Hazard ratio	95%CI†	P*	Hazard ratio	95%CI†	P*
PTBP3	0.549	0.401-0.751	<0.001	0.524	0.375-0.732	<0.001
Age	1.020	0.767-1.357	0.891	0.988	0.732-1.333	0.937
Gender	1.290	0.966-1.724	0.085	1.270	0.935-1.725	0.125
Tumor size	1.371	0.970-1.939	0.074	1.304	0.894-1.901	0.168
TNM stage	1.730	1.260-2.376	0.001	1.458	1.028-2.069	0.034

*Coding of variables: Cancer was coded as 1 (negative), and 2 (positive). Age was coded as 1 (≤ 56 years), and 2 (> 56 years). Gender was coded as 1 (male), and 2 (female).Tumor size was coded as 1 (< 7cm), and 2 (≥7 cm). TNM stage was coded as 1 (I-II), and 2 (III-IV).

†CI: confidence interval.

Table S3. Clinical samples from Shanghai Outdo Biotech Company related to Figure 1

Case	Age	Gender	Pathological diagnosis	Race
1	55	male	normal renal tissue	Asian (China)
2	70	female	normal renal tissue	Asian (China)
3	71	male	normal renal tissue	Asian (China)
4	54	male	normal renal tissue	Asian (China)
5	70	male	normal renal tissue	Asian (China)
6	70	male	normal renal tissue	Asian (China)
7	69	female	normal renal tissue	Asian (China)
8	79	male	normal renal tissue	Asian (China)
9	52	female	normal renal tissue	Asian (China)
10	55	male	normal renal tissue	Asian (China)
11	29	male	normal renal tissue	Asian (China)
12	57	male	normal renal tissue	Asian (China)
13	53	male	normal renal tissue	Asian (China)
14	59	male	normal renal tissue	Asian (China)
15	70	male	normal renal tissue	Asian (China)
16	51	female	normal renal tissue	Asian (China)
17	53	female	normal renal tissue	Asian (China)
18	43	female	normal renal tissue	Asian (China)
19	64	male	normal renal tissue	Asian (China)
20	58	male	normal renal tissue	Asian (China)

21	76	female	normal renal tissue	Asian (China)
22	42	male	normal renal tissue	Asian (China)
23	51	female	normal renal tissue	Asian (China)
24	74	male	normal renal tissue	Asian (China)
25	44	male	normal renal tissue	Asian (China)
26	54	male	normal renal tissue	Asian (China)
27	52	female	normal renal tissue	Asian (China)
28	69	male	normal renal tissue	Asian (China)
29	69	male	normal renal tissue	Asian (China)
30	63	female	normal renal tissue	Asian (China)
31	53	male	normal renal tissue	Asian (China)
32	56	female	normal renal tissue	Asian (China)
33	48	male	normal renal tissue	Asian (China)
34	52	female	normal renal tissue	Asian (China)
35	60	male	normal renal tissue	Asian (China)
36	57	male	normal renal tissue	Asian (China)
37	42	male	normal renal tissue	Asian (China)
38	42	male	normal renal tissue	Asian (China)
39	44	female	normal renal tissue	Asian (China)
40	72	female	normal renal tissue	Asian (China)
41	46	male	normal renal tissue	Asian (China)
42	82	male	normal renal tissue	Asian (China)

43	66	female	normal renal tissue	Asian (China)
44	57	female	normal renal tissue	Asian (China)
45	56	male	normal renal tissue	Asian (China)
46	53	male	normal renal tissue	Asian (China)
47	52	male	normal renal tissue	Asian (China)
48	50	male	normal renal tissue	Asian (China)
49	73	female	normal renal tissue	Asian (China)
50	62	male	normal renal tissue	Asian (China)
51	71	male	normal renal tissue	Asian (China)
52	57	female	normal renal tissue	Asian (China)
53	66	female	normal renal tissue	Asian (China)
54	57	male	normal renal tissue	Asian (China)
55	59	male	normal renal tissue	Asian (China)
56	56	male	normal renal tissue	Asian (China)
57	53	male	normal renal tissue	Asian (China)
58	73	male	normal renal tissue	Asian (China)
59	57	male	normal renal tissue	Asian (China)
60	48	female	normal renal tissue	Asian (China)
61	66	female	normal renal tissue	Asian (China)
62	80	male	normal renal tissue	Asian (China)
63	55	male	normal renal tissue	Asian (China)
64	65	male	normal renal tissue	Asian (China)

65	50	male	normal renal tissue	Asian (China)
66	61	male	normal renal tissue	Asian (China)
67	71	female	normal renal tissue	Asian (China)
68	55	male	normal renal tissue	Asian (China)
69	47	female	normal renal tissue	Asian (China)
70	67	female	normal renal tissue	Asian (China)
71	72	male	normal renal tissue	Asian (China)
72	56	male	normal renal tissue	Asian (China)
73	49	male	normal renal tissue	Asian (China)
74	54	male	normal renal tissue	Asian (China)
75	55	male	renal cancer tissue	Asian (China)
76	70	female	renal cancer tissue	Asian (China)
77	71	male	renal cancer tissue	Asian (China)
78	54	male	renal cancer tissue	Asian (China)
79	70	male	renal cancer tissue	Asian (China)
80	70	male	renal cancer tissue	Asian (China)
81	69	female	renal cancer tissue	Asian (China)
82	79	male	renal cancer tissue	Asian (China)
83	52	female	renal cancer tissue	Asian (China)
84	55	male	renal cancer tissue	Asian (China)
85	29	male	renal cancer tissue	Asian (China)
86	57	male	renal cancer tissue	Asian (China)

87	53	male	renal cancer tissue	Asian (China)
88	59	male	renal cancer tissue	Asian (China)
89	70	male	renal cancer tissue	Asian (China)
90	51	female	renal cancer tissue	Asian (China)
91	53	female	renal cancer tissue	Asian (China)
92	43	female	renal cancer tissue	Asian (China)
93	64	male	renal cancer tissue	Asian (China)
94	58	male	renal cancer tissue	Asian (China)
95	76	female	renal cancer tissue	Asian (China)
96	42	male	renal cancer tissue	Asian (China)
97	51	female	renal cancer tissue	Asian (China)
98	74	male	renal cancer tissue	Asian (China)
99	44	male	renal cancer tissue	Asian (China)
100	54	male	renal cancer tissue	Asian (China)
101	52	female	renal cancer tissue	Asian (China)
102	69	male	renal cancer tissue	Asian (China)
103	69	male	renal cancer tissue	Asian (China)
104	63	female	renal cancer tissue	Asian (China)
105	53	male	renal cancer tissue	Asian (China)
106	56	female	renal cancer tissue	Asian (China)
107	48	male	renal cancer tissue	Asian (China)
108	52	female	renal cancer tissue	Asian (China)

109	60	male	renal cancer tissue	Asian (China)
110	57	male	renal cancer tissue	Asian (China)
111	42	male	renal cancer tissue	Asian (China)
112	42	male	renal cancer tissue	Asian (China)
113	44	female	renal cancer tissue	Asian (China)
114	72	female	renal cancer tissue	Asian (China)
115	46	male	renal cancer tissue	Asian (China)
116	82	male	renal cancer tissue	Asian (China)
117	66	female	renal cancer tissue	Asian (China)
118	57	female	renal cancer tissue	Asian (China)
119	56	male	renal cancer tissue	Asian (China)
120	53	male	renal cancer tissue	Asian (China)
121	52	male	renal cancer tissue	Asian (China)
122	50	male	renal cancer tissue	Asian (China)
123	73	female	renal cancer tissue	Asian (China)
124	62	male	renal cancer tissue	Asian (China)
125	71	male	renal cancer tissue	Asian (China)
126	57	female	renal cancer tissue	Asian (China)
127	66	female	renal cancer tissue	Asian (China)
128	57	male	renal cancer tissue	Asian (China)
129	59	male	renal cancer tissue	Asian (China)
130	56	male	renal cancer tissue	Asian (China)

131	53	male	renal cancer tissue	Asian (China)
132	73	male	renal cancer tissue	Asian (China)
133	57	male	renal cancer tissue	Asian (China)
134	48	female	renal cancer tissue	Asian (China)
135	66	female	renal cancer tissue	Asian (China)
136	80	male	renal cancer tissue	Asian (China)
137	55	male	renal cancer tissue	Asian (China)
138	65	male	renal cancer tissue	Asian (China)
139	50	male	renal cancer tissue	Asian (China)
140	61	male	renal cancer tissue	Asian (China)
141	71	female	renal cancer tissue	Asian (China)
142	55	male	renal cancer tissue	Asian (China)
143	47	female	renal cancer tissue	Asian (China)
144	67	female	renal cancer tissue	Asian (China)
145	72	male	renal cancer tissue	Asian (China)
146	56	male	renal cancer tissue	Asian (China)
147	49	male	renal cancer tissue	Asian (China)
148	54	male	renal cancer tissue	Asian (China)

Table S4. Clinical samples from the Affiliated Hospital of Xuzhou Medical University related to Figure 1, Figure 6 and Table 1

Case	Gender	TNM Stage	Overall survival (month)	Disease free survival (month)	Death or not (1=death; 0=not)	Age	Race
1	male	2	1.5	1	1	35	Asian (China)
2	male	5	1.5	N/A	1	77	Asian (China)
3	female	4	1.5	1	1	43	Asian (China)
4	female	3	1.5	1	1	26	Asian (China)
5	male	3	2	1	1	75	Asian (China)
6	male	4	2	1	1	43	Asian (China)
7	male	3	2.5	1	1	74	Asian (China)
8	male	2	2.5	1	1	76	Asian (China)
9	male	4	2.5	1	1	45	Asian (China)
10	male	1	2.5	1	1	77	Asian (China)
11	male	3	2.5	1	1	47	Asian (China)
12	female	4	2.5	1	1	73	Asian (China)
13	male	1	3	1	1	74	Asian

							(China)
14	female	1	3	1	1	65	Asian (China)
15	female	4	3	1	1	46	Asian (China)
16	female	1	3	1	1	57	Asian (China)
17	female	2	3.5	1	1	54	Asian (China)
18	female	3	3.5	N/A	1	70	Asian (China)
19	male	1	4	1	1	31	Asian (China)
20	male	1	4.5	1	1	47	Asian (China)
21	male	3	4.5	N/A	1	77	Asian (China)
22	female	3	4.5	N/A	1	53	Asian (China)
23	male	2	5	1	1	63	Asian (China)
24	male	2	5	1	1	44	Asian (China)
25	male	4	5	1	1	6	Asian (China)
26	female	4	5	1	1	4	Asian (China)
27	female	2	5	1	1	65	Asian (China)
28	male	1	5.5	1	1	69	Asian (China)
29	female	3	5.5	1	1	68	Asian (China)
30	female	4	5.5	N/A	1	54	Asian (China)
31	male	2	6	1	1	61	Asian (China)
32	male	1	6	1	1	75	Asian (China)
33	female	2	8	1	1	41	Asian (China)
34	male	4	9	1	1	60	Asian (China)

35	female	4	9	1	1	57	Asian (China)
36	male	5	12.5	N/A	1	67	Asian (China)
37	male	5	13	1	1	63	Asian (China)
38	male	1	13	1	1	49	Asian (China)
39	male	1	14	1	1	60	Asian (China)
40	female	4	14.5	N/A	1	17	Asian (China)
41	female	3	15.5	N/A	1	48	Asian (China)
42	male	3	17	1	1	74	Asian (China)
43	female	2	17	1	1	65	Asian (China)
44	male	5	17.5	N/A	1	65	Asian (China)
45	male	1	18	1	1	67	Asian (China)
46	male	3	18.5	1	1	63	Asian (China)
47	male	1	18.5	N/A	1	70	Asian (China)
48	female	5	18.5	N/A	1	45	Asian (China)
49	female	1	19	1	1	74	Asian (China)
50	male	5	19.5	N/A	1	67	Asian (China)
51	female	3	19.5	N/A	1	11	Asian (China)
52	male	3	20.5	N/A	1	52	Asian (China)
53	male	4	21.5	N/A	1	50	Asian (China)
54	male	1	23	1	1	45	Asian (China)
55	female	1	23.5	N/A	1	57	Asian (China)
56	male	3	24.5	1	1	43	Asian (China)

57	male	1	24.5	N/A	1	58	Asian (China)
58	female	1	24.5	1	1	58	Asian (China)
59	male	1	25	1	1	35	Asian (China)
60	male	1	26	1	1	62	Asian (China)
61	male	5	26	1	1	34	Asian (China)
62	female	1	26	1	1	59	Asian (China)
63	male	4	26.5	1	1	55	Asian (China)
64	male	5	26.5	1	1	46	Asian (China)
65	male	2	27.5	N/A	1	74	Asian (China)
66	male	2	28.5	1	1	54	Asian (China)
67	male	4	28.5	1	1	58	Asian (China)
68	male	2	28.5	1	1	66	Asian (China)
69	female	1	28.5	1	1	56	Asian (China)
70	male	2	29.5	1	1	84	Asian (China)
71	female	3	29.5	1	1	46	Asian (China)
72	female	1	29.5	1	1	46	Asian (China)
73	female	1	29.5	1	1	70	Asian (China)
74	female	1	29.5	1	1	61	Asian (China)
75	male	3	30	1	1	48	Asian (China)
76	male	1	30.5	1	1	62	Asian (China)
77	male	1	30.5	1	1	58	Asian (China)
78	female	1	30.5	1	1	57	Asian (China)

79	female	2	30.5	1	1	50	Asian (China)
80	female	1	30.5	1	1	72	Asian (China)
81	female	3	30.5	1	1	59	Asian (China)
82	female	3	31	1	1	40	Asian (China)
83	male	1	31.5	1	1	51	Asian (China)
84	male	5	31.5	1	1	76	Asian (China)
85	male	1	31.5	1	1	42	Asian (China)
86	male	1	31.5	1	1	49	Asian (China)
87	male	1	31.5	1	1	46	Asian (China)
88	male	1	32.5	1	1	64	Asian (China)
89	male	1	32.5	1	1	68	Asian (China)
90	male	1	32.5	1	1	41	Asian (China)
91	female	2	32.5	1	1	55	Asian (China)
92	female	1	32.5	1	1	27	Asian (China)
93	female	1	32.5	1	1	46	Asian (China)
94	female	1	32.5	1	1	44	Asian (China)
95	female	1	32.5	1	1	70	Asian (China)
96	male	2	33.5	1	1	57	Asian (China)
97	male	1	33.5	1	1	52	Asian (China)
98	male	1	33.5	1	1	65	Asian (China)
99	male	1	34	1	1	40	Asian (China)
100	female	1	34	1	1	46	Asian (China)

101	female	1	34	1	1	67	Asian (China)
102	male	2	34.5	1	1	56	Asian (China)
103	male	1	35.5	1	1	67	Asian (China)
104	male	1	35.5	1	1	59	Asian (China)
105	male	1	35.5	1	1	68	Asian (China)
106	female	1	35.5	1	1	61	Asian (China)
107	male	1	36	1	1	44	Asian (China)
108	male	3	36.5	1	1	86	Asian (China)
109	female	3	37	1	1	63	Asian (China)
110	male	3	37.5	1	1	62	Asian (China)
111	male	1	37.5	1	1	40	Asian (China)
112	female	4	37.5	1	1	67	Asian (China)
113	female	3	37.5	1	1	41	Asian (China)
114	female	1	37.5	1	1	82	Asian (China)
115	male	1	38	1	1	80	Asian (China)
116	male	1	38.5	1	1	77	Asian (China)
117	female	3	38.5	1	1	44	Asian (China)
118	female	1	38.5	1	1	38	Asian (China)
119	male	1	39	1	1	44	Asian (China)
120	male	1	39	1	1	68	Asian (China)
121	male	1	39.5	1	1	52	Asian (China)
122	male	1	39.5	1	1	77	Asian (China)

123	male	1	39.5	N/A	1	67	Asian (China)
124	male	1	40	1	1	61	Asian (China)
125	male	1	40	1	1	51	Asian (China)
126	male	1	40	1	1	48	Asian (China)
127	male	2	41.5	1	1	52	Asian (China)
128	male	5	41.5	1	1	61	Asian (China)
129	female	1	41.5	1	1	72	Asian (China)
130	female	2	41.5	1	1	84	Asian (China)
131	female	1	41.5	1	1	63	Asian (China)
132	male	1	42.5	1	1	48	Asian (China)
133	male	1	42.5	1	1	58	Asian (China)
134	female	5	42.5	1	1	72	Asian (China)
135	female	1	42.5	1	1	32	Asian (China)
136	male	3	43.5	1	1	53	Asian (China)
137	male	3	43.5	1	1	46	Asian (China)
138	male	1	43.5	1	1	51	Asian (China)
139	male	3	43.5	1	1	55	Asian (China)
140	male	1	43.5	1	1	49	Asian (China)
141	female	1	43.5	1	1	51	Asian (China)
142	male	2	44	1	1	53	Asian (China)
143	male	1	44	1	1	48	Asian (China)
144	male	1	44.5	1	1	66	Asian (China)

145	male	1	44.5	1	1	52	Asian (China)
146	female	3	44.5	1	1	47	Asian (China)
147	female	3	44.5	1	1	47	Asian (China)
148	female	1	44.5	1	1	52	Asian (China)
149	male	1	45.5	1	1	58	Asian (China)
150	female	1	45.5	1	1	53	Asian (China)
151	male	1	46	1	1	41	Asian (China)
152	male	2	46.5	1	1	70	Asian (China)
153	female	3	46.5	1	1	70	Asian (China)
154	female	1	47	1	1	62	Asian (China)
155	male	2	47.5	1	1	43	Asian (China)
156	male	1	47.5	1	1	65	Asian (China)
157	male	1	47.5	1	1	51	Asian (China)
158	male	1	47.5	1	1	70	Asian (China)
159	male	1	47.5	N/A	1	75	Asian (China)
160	female	1	47.5	1	1	44	Asian (China)
161	female	1	47.5	1	1	70	Asian (China)
162	male	1	48	1	1	72	Asian (China)
163	male	3	48.5	1	1	45	Asian (China)
164	female	1	48.5	1	1	49	Asian (China)
165	male	1	49.5	1	1	42	Asian (China)
166	male	4	50.5	1	1	58	Asian (China)

167	male	5	50.5	1	1	70	Asian (China)
168	male	1	50.5	N/A	1	63	Asian (China)
169	female	1	50.5	1	1	56	Asian (China)
170	male	1	51.5	1	1	71	Asian (China)
171	male	1	51.5	1	1	37	Asian (China)
172	male	1	51.5	1	1	39	Asian (China)
173	male	1	52.5	1	1	68	Asian (China)
174	male	1	52.5	1	1	36	Asian (China)
175	male	1	52.5	1	1	37	Asian (China)
176	male	1	52.5	1	1	59	Asian (China)
177	male	1	52.5	1	1	65	Asian (China)
178	female	1	52.5	1	1	41	Asian (China)
179	male	1	53.5	1	1	70	Asian (China)
180	female	1	53.5	1	1	35	Asian (China)
181	female	2	53.5	1	1	69	Asian (China)
182	female	3	53.5	1	1	59	Asian (China)
183	female	1	54.5	1	1	51	Asian (China)
184	female	1	54.5	1	1	60	Asian (China)
185	female	1	54.5	1	1	50	Asian (China)
186	male	5	55	1	1	50	Asian (China)
187	male	2	55.5	1	1	58	Asian (China)
188	male	1	56.5	1	1	60	Asian (China)

189	male	3	56.5	1	1	56	Asian (China)
190	male	1	56.5	1	1	60	Asian (China)
191	female	1	56.5	1	1	32	Asian (China)
192	male	1	57.5	1	1	80	Asian (China)
193	female	1	57.5	1	1	67	Asian (China)
194	male	1	58.5	1	1	63	Asian (China)
195	male	1	58.5	1	1	31	Asian (China)
196	female	1	58.5	1	1	61	Asian (China)
197	female	5	58.5	1	1	54	Asian (China)
198	male	5	59.5	1	1	66	Asian (China)
199	male	2	59.5	1	1	59	Asian (China)
200	male	3	59.5	1	1	58	Asian (China)
201	male	1	60	0	0	55	Asian (China)
202	male	2	60	0	0	65	Asian (China)
203	male	1	60	0	0	78	Asian (China)
204	male	1	60	0	0	43	Asian (China)
205	male	1	60	0	0	36	Asian (China)
206	male	2	60	0	0	78	Asian (China)
207	male	1	60	0	0	51	Asian (China)
208	male	1	60	0	0	63	Asian (China)
209	male	1	60	0	0	35	Asian (China)
210	male	1	60	0	0	61	Asian (China)

211	male	5	60	0	0	43	Asian (China)
212	male	1	60	0	0	70	Asian (China)
213	male	1	60	0	0	49	Asian (China)
214	male	5	60	0	0	53	Asian (China)
215	male	1	60	0	0	46	Asian (China)
216	male	5	60	0	0	46	Asian (China)
217	male	2	60	0	0	67	Asian (China)
218	male	5	60	0	0	57	Asian (China)
219	male	5	60	0	0	55	Asian (China)
220	male	1	60	0	0	61	Asian (China)
221	male	1	60	0	0	65	Asian (China)
222	male	1	60	0	0	58	Asian (China)
223	male	3	60	0	0	73	Asian (China)
224	male	1	60	0	0	74	Asian (China)
225	male	1	60	0	0	62	Asian (China)
226	male	1	60	0	0	69	Asian (China)
227	male	1	60	0	0	54	Asian (China)
228	male	1	60	0	0	40	Asian (China)
229	male	1	60	0	0	38	Asian (China)
230	male	1	60	0	0	46	Asian (China)
231	male	1	60	0	0	48	Asian (China)
232	female	1	60	0	0	44	Asian (China)

233	female	2	60	0	0	18	Asian (China)
234	female	1	60	0	0	57	Asian (China)
235	female	1	60	0	0	57	Asian (China)
236	female	1	60	0	0	46	Asian (China)
237	female	1	60	0	0	55	Asian (China)
238	female	1	60	0	0	63	Asian (China)
239	female	1	60	0	0	54	Asian (China)
240	female	1	60	0	0	70	Asian (China)
241	female	1	60	0	0	50	Asian (China)
242	female	2	60	0	0	40	Asian (China)
243	female	1	60	0	0	60	Asian (China)
244	male	4	N/A	N/A	N/A	61	Asian (China)
245	male	5	N/A	N/A	N/A	34	Asian (China)
246	male	1	N/A	N/A	N/A	80	Asian (China)
247	male	4	N/A	N/A	N/A	61	Asian (China)
248	male	2	N/A	N/A	N/A	47	Asian (China)
249	male	1	N/A	N/A	N/A	46	Asian (China)
250	male	2	N/A	N/A	N/A	55	Asian (China)
251	male	1	N/A	N/A	N/A	56	Asian (China)
252	male	1	N/A	N/A	N/A	47	Asian (China)
253	male	1	N/A	N/A	N/A	73	Asian (China)
254	male	5	N/A	N/A	N/A	67	Asian (China)

255	male	1	N/A	N/A	N/A	78	Asian (China)
256	male	3	N/A	N/A	N/A	3	Asian (China)
257	male	2	N/A	N/A	N/A	58	Asian (China)
258	male	2	N/A	N/A	N/A	50	Asian (China)
259	male	5	N/A	N/A	N/A	53	Asian (China)
260	male	1	N/A	N/A	N/A	54	Asian (China)
261	male	2	N/A	N/A	N/A	73	Asian (China)
262	male	1	N/A	N/A	N/A	48	Asian (China)
263	male	5	N/A	N/A	N/A	58	Asian (China)
264	male	1	N/A	N/A	N/A	63	Asian (China)
265	male	2	N/A	N/A	N/A	40	Asian (China)
266	male	5	N/A	N/A	N/A	67	Asian (China)
267	male	1	N/A	N/A	N/A	43	Asian (China)
268	male	5	N/A	N/A	N/A	39	Asian (China)
269	male	1	N/A	N/A	N/A	76	Asian (China)
270	male	1	N/A	N/A	N/A	39	Asian (China)
271	male	4	N/A	N/A	N/A	20	Asian (China)
272	male	2	N/A	N/A	N/A	72	Asian (China)
273	male	1	N/A	N/A	N/A	49	Asian (China)
274	male	3	N/A	N/A	N/A	53	Asian (China)
275	male	1	N/A	N/A	N/A	49	Asian (China)
276	male	5	N/A	N/A	N/A	67	Asian (China)

277	male	1	N/A	N/A	N/A	61	Asian (China)
278	male	1	N/A	N/A	N/A	38	Asian (China)
279	male	5	N/A	N/A	N/A	65	Asian (China)
280	male	1	N/A	N/A	N/A	60	Asian (China)
281	male	5	N/A	N/A	N/A	65	Asian (China)
282	male	3	N/A	N/A	N/A	58	Asian (China)
283	male	1	N/A	N/A	N/A	57	Asian (China)
284	male	1	N/A	N/A	N/A	59	Asian (China)
285	male	1	N/A	N/A	N/A	58	Asian (China)
286	male	1	N/A	N/A	N/A	68	Asian (China)
287	male	1	N/A	N/A	N/A	39	Asian (China)
288	female	2	N/A	N/A	N/A	65	Asian (China)
289	female	1	N/A	N/A	N/A	51	Asian (China)
290	female	1	N/A	N/A	N/A	60	Asian (China)
291	female	3	N/A	N/A	N/A	60	Asian (China)
292	female	2	N/A	N/A	N/A	62	Asian (China)
293	female	1	N/A	N/A	N/A	73	Asian (China)
294	female	3	N/A	N/A	N/A	56	Asian (China)
295	female	1	N/A	N/A	N/A	66	Asian (China)
296	female	1	N/A	N/A	N/A	39	Asian (China)
297	female	2	N/A	N/A	N/A	49	Asian (China)
298	female	1	N/A	N/A	N/A	48	Asian (China)

299	female	2	N/A	N/A	N/A	53	Asian (China)
300	female	5	N/A	N/A	N/A	72	Asian (China)
301	female	1	N/A	N/A	N/A	71	Asian (China)
302	female	1	N/A	N/A	N/A	50	Asian (China)

Table S5. Sequences used for shRNAs and siRNAs, related to STAR Methods

**Targets**	**Sequences(5'-3')**
shPTBP3#1	CCGGACCAGGAAATTCTGTTCTACTCTCGAGAGTAGAACAGAATTTCCTGGTTTTTG
shPTBP3#2	CCGGCAGAGACTTCACTCGCTTAGACTCGAGTCTAAGCGAGTGAAGTCTCTGTTTTTG
siPTBP3	CCCAGUAAAUGCACAUUAUTT
siIGF2BP3	CACCUUGAAAGUAGCCUAUTT

Table S6. Primers used for qRT-PCR, related to STAR Methods

**Gene**	**Sequence(5'-3')**
GAPDH-For	AAGGTCGGAGTCAACGGATTTG
GAPDH-Rev	CCATGGGTGGAATCATATTGGAA
PTBP3-For	ACAGCTAATGGGAATGACAGCA
PTBP3-Rev	CTGGCTTCGAAGGTGAGGAG
E-cad-For	GACAACAAGCCCGAATT
E-cad-Rev	GGAAACTCTCTCGGTCCA
N-cad-For	CGGGTAATCCTCCCAAATCA
N-cad-Rev	CTTTATCCCGGCGTTTCATC
FN-For	CAGTGGGAGACCTCGAGAAG
FN-Rev	TCCCTCGGAACATCAGAAAC

HMGA1-For	AGCGAAGTGCCAACACCTAAG
HMGA1-Rev	TGGTGGTTTTCCGGGTCTTG
18SrRNA-For	GTAACCCGTTGAACCCCATT
18SrRNA-Rev	CCATCCAATCGGTAGTAGCG
